# Population pharmacokinetic analysis of nanoparticle-bound and free camptothecin after administration of NLG207 in adults with advanced solid tumors

**DOI:** 10.1007/s00280-020-04134-9

**Published:** 2020-09-08

**Authors:** Keith T. Schmidt, Alwin D. R. Huitema, Thomas P. C. Dorlo, Cody J. Peer, Lisa M. Cordes, Linda Sciuto, Susan Wroblewski, Yves Pommier, Ravi A. Madan, Anish Thomas, William D. Figg

**Affiliations:** 1 Clinical Pharmacology Program, Center for Cancer Research, National Cancer Institute, National Institutes of Health, 9000 Rockville Pike, Building 10/Room 5A03, Bethesda, MD 20892 USA; 2 Department Pharmacy and Pharmacology, Netherlands Cancer Institute, Amsterdam, The Netherlands; 3 Department of Clinical Pharmacy, University Medical Center Utrecht, Utrecht University, Utrecht, The Netherlands; 4 Genitourinary Malignancies Branch, Center for Cancer Research, National Cancer Institute, National Institutes of Health, Bethesda, MD USA; 5 Developmental Therapeutics Branch, Center for Cancer Research, National Cancer Institute, National Institutes of Health, Bethesda, MD USA

**Keywords:** Nanoparticle, Drug release, Population pharmacokinetics, Topoisomerase I, NLMEM

## Abstract

**Purpose:**

NLG207 (formerly CRLX101) is a nanoparticle–drug conjugate (NDC) of the potent topoisomerase I inhibitor, camptothecin (CPT). The present study sought to characterize the complex pharmacokinetics (PK) of NLG207 and better describe CPT release from nanoparticles using a population PK (popPK) model.

**Methods:**

From 27 patients enrolled on two phase II clinical trials (NCT02769962 and NCT03531827), dense sampling was performed up to 48 h post-administration of NLG207 during cycle one and six of treatment; samples were also collected at ~ 360 h post-dose. Conjugated and free CPT concentrations were quantified from each sample, resulting in 477 observations to build a popPK model using non-linear mixed-effects modeling.

**Results:**

The PK of NLG207 was characterized by combining two linear two-compartment models with first-order kinetics each to describe nanoparticle-bound (conjugated) and free CPT. Allometric scaling based on body weight provided the best body-size descriptor for all PK parameters. The typical volumes of distribution of the conjugated CPT central and free CPT central compartments were 3.16 L (BSV CV%; 18.1%) and 21.1 L (CV%; 79.8%), respectively. CPT release from the nanoparticle formulation was characterized via an initial rapid clearance of 5.71 L/h (CV%; 62.6%), which decreased via first-order decay (estimated half-life of 0.307 h) to the steady-state value of 0.0988 L/h (CV%; 33.5%) by ~ 4 h after end of infusion. Renal clearance of free CPT was 0.874 L/h (CV%; 42.2%).

**Conclusion:**

The popPK model confirmed nanoparticle behavior of conjugated CPT and mechanistically characterized CPT release from NLG207. The current analysis provides a strong foundation for future study as a potential predictive tool in ongoing NLG207 clinical trials.

**Electronic supplementary material:**

The online version of this article (10.1007/s00280-020-04134-9) contains supplementary material, which is available to authorized users.

## Introduction

NLG207 (formerly CRLX101) is a nanoparticle–drug conjugate (NDC) of camptothecin (CPT), a potent topoisomerase I (TOP1) inhibitor, designed to overcome the poor physicochemical and pharmacokinetic (PK) properties (e.g., pH labile activation, limited plasma solubility) associated with small molecule delivery to tumors [1]. Despite significant activity in preclinical models, CPT showed minimal anti-tumor activity in a clinical setting, and was associated with significant toxicities, primarily myelosuppression and hemorrhagic cystitis [2–5]. Current FDA-approved TOP1 inhibitors (e.g., irinotecan and topotecan) are derived from the CPT backbone with improved solubility, but are still associated with sub-optimal PK and clinically significant toxicity [6]. NLG207 consists of co-polymer units of β-cyclodextrin linked to adjacent units of poly-ethylene glycol (PEG), with conjugation of CPT via carboxylate esters. In aqueous solution, 4–5 co-polymer strands readily interact and fold into nanoparticles of 20–40 nm in diameter, providing a soluble delivery vehicle with increased plasma retention time [7, 8]. The properties of the nanoparticle reduce glomerular filtration, mononuclear phagocyte system (MPS) interactions, and uptake into healthy tissues, while enabling passive selection into tumor via an enhanced permeation and retention (EPR) effect driven by size exclusion [8]. Once deposited into tumors, CPT is slowly released from the NDCs via pH-dependent hydrolysis of the carboxylate esters and subsequently renally cleared, limiting exposure of the small molecule in the systemic circulation [7].

NLG207 is well tolerated, with over 300 patients spanning multiple tumor histologies (e.g., non-small-cell lung cancer, advanced ovarian cancer, and metastatic renal cell carcinoma) receiving at least one dose via numerous clinical trials [9–14]. In the majority of studies, NLG207 has been administered at a dose of 15 mg/m^2^ via intravenous infusion every 2 weeks, with once weekly and 12 mg/m^2^ dosing strategies also investigated [1]. TOP1 inhibitors also have HIF-1α modulating effects [15], leading to evaluation of NLG207 as both monotherapy, and in combination with either a secondary chemotherapeutic agent (e.g., paclitaxel) or anti-angiogenic agent (e.g., bevacizumab) [10, 13, 16]. Ongoing studies at the National Cancer Institute (NCI) are evaluating the efficacy of NLG207 in combination with the PARP inhibitor, olaparib, in small-cell lung cancer, urothelial carcinoma, and prostate cancer, and with the androgen receptor antagonist, enzalutamide, in prostate cancer (NCT02769962 and NCT03531827, respectively).

Despite improvements in CPT delivery, commonly reported adverse events (AEs) of NLG207 included fatigue, myelosuppression, and, notably, bladder-associated toxicity [9, 10, 12–14]. In response to hemorrhagic cystitis occurring early in phase I dose escalation, hydration strategies have been successfully implemented pre- and post-NLG207 infusion to dilute accumulated CPT concentrations in the bladder; however, a low frequency of subsequent patients receiving NLG207 still reported low-grade cystitis, dysuria, and hematuria [14]. Variable urinary excretion of CPT during the first 48 h post-infusion has also been reported, with a mean of 21% of the total NLG207 dose eliminated in urine, a majority of which is eliminated in the first 8 h [14]. Previous non-compartmental analyses of NLG207 PK, which have analyzed nanoparticle-bound (conjugated) and free CPT independently, did not adequately address the release of CPT from the formulation, a critical component to understanding free CPT exposure and potential correlation to clinically observed toxicity.

The purpose of the present population pharmacokinetic (popPK) analysis was to 1) better understand CPT release from the NLG207 NDCs following administration, and 2) fully characterize the complex PK of conjugated and free CPT via a harmonized model.

## Methods

### Patients and study design

NLG207 PK were evaluated using a population of patients with advanced solid tumors enrolled in two clinical studies at the National Cancer Institute analyzing NLG207 in combination with either olaparib or enzalutamide (NCT02769962 and NCT03531827, respectively). The PK analyses of these studies were designed to address potential drug–drug interactions associated with co-administration of olaparib or enzaluamide; the popPK analyses are a post hoc study objective. Both studies were conducted according to IRB-approved protocols and all patients provided written informed consent.

NLG207 was administered via 12 mg/m^2^ intravenous infusions over 1 or 2 h every two weeks, with the potential for incremental dose changes (e.g., decrease to 9 or 6 mg/m^2^) in subsequent cycles depending on the study and tolerability. Blood samples were collected during cycle 1 for both studies without co-administration of either olaparib or enzalutamide. Collection of samples occurred up to 48 h (pre-dose, mid-infusion [MI], end-of infusion [EOI], 1, 2, 12, 24 and 48 h post-EOI), and up to 24 h (pre-dose, MI, EOI, 1, 2, 3, 4, 8, 12, and 24 h post-EOI) for NCT02769962 and NCT03531827, respectively. For NCT02769962, olaparib was only administered 48 h after completion of the NLG207 infusion and administration was discontinued 48 h prior to subsequent NLG207 infusions, reducing the likelihood of compounded myelosuppression from both agents in combination [17]; additional samples were collected ~ 336 to 384 h (14–16 days) post-EOI and during cycle 6 using the same timepoints for patients remaining on therapy. Data collected on NCT03531827 only include samples from cycle 1. Of note, NLG207 on NCT02776992 was administered at a slower rate during the first 10–15 min infusion, before increasing the rate to complete the infusion over the course of 1 or 2 h, whereas NCT03531827 administered NLG207 at a constant infusion rate over the course of 1 h.

### Pharmacokinetic samples and analyses

Blood samples, collected in sodium heparin treated tubes (green top tubes, BD Biosciences), were processed into plasma via a standard protocol using a refrigerated centrifuge, and plasma was stored at − 80 °C until the determination of CPT concentrations. Samples were assayed for the quantitation of conjugated and free CPT concentrations using a validated assay involving liquid chromatography (LC) with tandem mass spectrometric (MS/MS) detection, as previously described [1]. Quantitation of all samples was within the pre-specified calibration ranges (10–10,000 ng/mL and 1–1000 ng/mL for total and free CPT, respectively), with acceptable quantitation meeting FDA guidance criteria for precision and accuracy of ≤ 15% relative error [18]. Nanoparticle-bound CPT concentrations were determined via the subtraction of the free CPT quantitation from the total CPT quantitation: conjugated and free CPT were utilized for observations in the development of this model.

A population approach based on non-linear mixed-effects modeling using the software package NONMEM version 7.4.3 (Icon, Hanover, MD, USA) was applied. Pirana version 2.9.9, PsN version 4.9, and R version 3.6.1 were used for workflow management, data handling, and data visualization, respectively [19]. The first-order conditional estimation option with interaction between random and residual error components (FOCE-I), as implemented in NONMEM, was used as the estimation method.

### Pharmacokinetic model-building procedure

Models were generated using custom differential equations via the ADVAN13 subroutine. At the start of structural model development, conjugated CPT disposition was evaluated separately using one-, two-, and three-compartment models with first-order kinetics. After fitting a linear two-compartment model to describe conjugated CPT, free CPT was incorporated into the model via testing of separate one-, two-, and three-compartment models with first-order kinetics (selection based on objective function value improvement and goodness-of-fit plots). Clearance terms to characterize transfer between conjugated and free CPT compartments and clearance of CPT from the model were also evaluated. Since the fraction of conjugated CPT converted to free CPT is unknown, all parameter estimates of free CPT were estimated relative to this fraction. For the model development of free CPT, we assumed that CPT release from the nanoparticle formulation only occurs in the central compartment of conjugated CPT. Accumulation of NLG207 nanoparticles in tumor tissue would yield pH-dependent release of CPT; however, within the first 48 h post-dose, the release rate in (tumor) tissue would be negligible with respect to the rate observed in plasma; at the physiological pH of 7.4, the CPT release rate is faster in comparison to that of (tumor) tissue, presumed to have pH values of 5–6 [7, 20, 21].

Between-subject variability (BSV) following a log-normal distribution was implemented into the model:

1 $${\theta }_{i}= {\theta }_{\mathrm{pop}}\times {e}^{{\eta }_{i}}$$

where *θ*_*i*_ represents the individual (post-hoc) value of the parameter for the *i*th individual, *θ*_pop_ represents the population mean for the parameter, and η_*i*_ predicts the empirical Bayesian estimate for BSV of *i*th individual, sampled from a normal distribution with a mean of 0 and a variance of *ω*^2^. Between occasion variability (BOV) was not considered in this model, as only five patients had samples for two cycles of treatment.

Residual error was determined separately for both conjugated and free CPT observations, assessed via incorporation of proportional error, additive error, or combination:

2 $${C}_{i,j,\mathrm{obs}} = {C}_{i,j,\mathrm{pred}}\times \left(1+{\varepsilon }_{\mathrm{proportional}}\right)+ {\varepsilon }_{\mathrm{additive}}$$

where *C*_*i,j*_ is the observed or predicted value for subject *i* at time *j*, *ε*_proportional_ is the proportional error component, and *ε*_additive_ is the additive error component. Residual error components are sampled from a normal distribution with a mean of zero and a variance of *σ*. Combination error models were preferred in the development of this model.

### Covariate model

A limited set of potential predictors (covariates) for variability in PK parameters were assessed following structural and stochastic PK model development. Evaluated covariates included patient-related (e.g., actual body weight [BW], age, and renal function) and treatment-related (e.g., cycle of treatment) factors. Continuous and binary categorical covariates were investigated using the following equations, respectively:

3 $${\theta }_{i}= {\theta }_{\mathrm{pop}}\times {\left(\frac{{\mathrm{cov}}_{i}}{{\mathrm{cov}}_{\mathrm{med}}}\right)}^{{\theta }_{\mathrm{cov}}}\times {e}^{{\eta }_{i}}$$

4 $${\theta }_{i}={\theta }_{\mathrm{pop}}\times {{\theta }_{\mathrm{cov}}}^{{\mathrm{cov}}_{i}}\times {e}^{{\eta }_{i}}$$

where *θ*_cov_ is the parameter estimate for the specified covariate, cov_*i*_ is the covariate value for the *i*th individual, and cov_med_ represents the median, or typical, value for the covariate in the population. The influence of body weight (BW) on PK parameters was either estimated or implemented via standard allometric scaling, with fixed exponents of 1 and 0.75 for volume of distribution and clearance terms, respectively, using Eq. 3; a more involved evaluation of allometric scaling beyond traditional implementation was done to (1) determine potential differences in the effect of body weight on conjugated CPT versus free CPT and (2) to determine if clearance terms representative of pH-dependent drug release were influenced by body weight.

### Pharmacokinetic model evaluation

Estimates of the structural and covariate model were deemed relevant only if scientifically and biologically plausible. Covariates were plotted against empirical Bayes estimates of BSV to elucidate potential parameter–covariate relationships. Addition of one parameter in hierarchical models was evaluated on the basis of objective function value (OFV), which is equal to minus twice the log-likelihood and assumed to follow a Chi-square distribution; a change in OFV (ΔOFV) of − 3.84 corresponded to a *p* value of 0.05 (i.e., one degree of freedom). Forward inclusion and backward elimination of a parameter required a significance level *p* < 0.005 (− 7.9 ΔOFV) and *p* < 0.001 (− 10.8 ΔOFV), respectively.

Standard goodness-of-fit (GOF) plots were generated to assess the model’s ability to appropriately characterize the data. These plots included observed concentrations plotted against population and individual-predicted concentrations, and conditional weighted residuals (CWRES) versus time and predicted concentrations. To further assess the value of model predictions, visual predictive checks (VPCs) were created to compare distributions of the simulated observations to real data observations. GOF and VPCs for conjugated and free CPT were evaluated independently, with separate plots generated for each set of observations.

Estimations of parameter uncertainty were obtained using the sampling importance resampling (SIR) method, an optimal approach to assess models with small datasets [22]. Initial proposal density was approximated in the absence of reliable $COVARIANCE output, with relative standard error (RSE) set to 25, 40, and 25 for THETA, OMEGA, and SIGMA parameters, respectively. The SIR method was run for six iterations with samples set to 5000 for each iteration and an increasing number of resamples for each iteration until iteration 4 (i.e., 200, 400, 500, 1000, 1000, and 1000). Correlations between parameter estimates were evaluated to ensure identifiability of each parameter specified and 95% confidence intervals (CI) for each parameter was reported in addition to RSE estimates, frequently with asymmetric CI. Values below 30% for shrinkage (BSV and residual error) were deemed acceptable.

## Results

### Patients and samples

A total of 27 patients with a median age of 60 were included in this study (Table 1). All patients received a 12 mg/m^2^ dose of NLG207 at the start of cycle one of treatment. Five patients received cycle 6 day 1 treatment, including two patients who had dose reductions (50% and 75%). In total, 239 total plasma samples collected over 32 total doses of NLG207 were included in the analysis. Each sample was quantitated for conjugated and free CPT, resulting in 477 total observations, with the omission of one observation due to incorrect sample handling. In samples collected within the first 3 h post-start of infusion, the observed average percent free CPT was 3.13  ±  0.21%; incremental increases in average percent free CPT occurred throughout the dosing interval, with a reported value of 20.42  ±  0.44% by approximately 50 h post-dose. None of the observations included were below or above the limit of quantitation for the validated assay.

**Table 1:** Patient characteristics

	*N* = 27 (%)
General characteristics	
Median age in years	60 (47–76)^&^
Median body weight in kg	70.4 (46.4–105)^&^
Gender	
Female	15 (55.6)
Male	12 (44.4)
Race	
African	4 (14.8)
Asian	3 (11.1)
Caucasian	20 (74.1)
Tumor type	
Non–small cell lung cancer	4 (14.8)
Small cell cancer	3 (11.1)
Pancreatic adenocarcinoma	3 (11.1)
Cholangiocarcinoma	3 (11.1)
Ovarian/fallopian tube cancer	3 (11.1)
Prostate cancer (mCRPC)^$^	3 (11.1)
Cervical cancer	2 (7.4)
Colorectal cancer	2 (7.4)
Mesothelioma	2 (7.4)
Myxofibrosarcoma	1 (3.7)
Thymic cancer	1 (3.7)
Renal function	
Mild renal impairment (eGFR = 60–90 ml/min/1.73 m^2^)	13 (48.1)
Normal renal function (eGFR > 90 ml/min/1.73 m^2^)	14 (51.9)
Presence of proteinuria	5 (18.5)
NCT02769962 (NLG207 + olaparib)	
Total	24 (88.9)
Cycle 1 PK collection Only	19 (79.9)^#^
Cycle 1 + Cycle 6 PK collection	5 (20.1)^#^
NCT03531827 (NLG207 + enzalutamide)	
Total	3 (11.1)
Cycle 1 PK collection Only	3 (100)^#^

*eGFR* estimated glomerular filtration rate

^&^Range of data reported

^$^All three prostate cancer patients were enrolled on NCT03531827

^#^Percentage of subgroup data within the selected population

### Structural model development

Disposition of conjugated CPT alone was first evaluated using linear one-, two-, and three-compartment models with first-order kinetics. The two-compartment and three-compartment models provided significant improvements over the one-compartment model, where the three-compartment model provided the lowest OFV. Incorporation of free CPT in the model was initially performed with the two-compartment conjugated CPT model, with comparisons to the three-compartment model to occur later in model development. BW was included as a covariate using standard allometric scaling, which reduced OFV by 70.56 units at this stage.

**Fig. 1:**
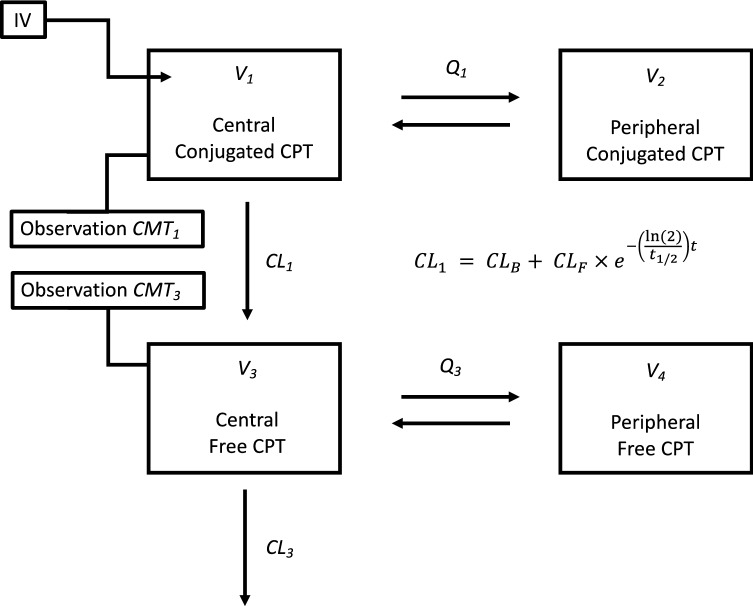
Overview of the four-compartment model describing disposition of nanoparticle-bound (conjugated) and free CPT, and the associated calculated parameters. Plasma sample quantitation enabled *CMT*_*1*_ and *CMT*_*3*_ observations for conjugated and free CPT concentrations, respectively. The model can be divided into two portions: conjugated CPT distribution described via estimates *V*_*1*_, *V*_*2*_, and *Q*_*1*_, and free CPT distribution described via estimates *V*_*3*_, *V*_*4*_, and *Q*_*3*_. *CL*_*1*_, or the conversion of conjugated to free CPT, was described using two additive clearance terms: *CL*_*B*_ is the “base” rate of conversion, and *CL*_*F*_ is a “fast” clearance rate that is modified via a simulated first-order decay term with estimated half-life, *t*_*1/2*_

Next, conversion of CPT from conjugated to free states was addressed. Conversion of CPT was first modeled using a first-order clearance term (CL_B_), which resulted in underprediction of unconjugated concentrations at early timepoints after infusion. It was hypothesized that the initial release rate of CPT from the nanoparticles was higher, relating to fast release of CPT molecules from the outer surface of the nanoparticle. Gradually, this release rate will lower and reach steady state. This was modeled with an initial fast clearance term (CL_F_), which showed a first-order decrease over time eventually reaching the slow steady-state clearance (CL_B_). The half-life (*t*_*1/2*_) of the fast clearance component was estimated to be 0.307 h, indicating a very rapid decrease in the release rate of CPT from the nanoparticles over time. Incorporating both terms for *CL*_*1*_ provided an increase in fit with a ΔOFV of -325 units. The final model is schematically depicted in Fig. 1.

Following the fit of parameters associated with CPT release, the three-compartmental model of conjugated CPT no longer provided a better fit of the data, justifying use of the two-compartment conjugated CPT model. The free CPT component of the model was then assessed via linear one-, two-, and three-compartment models. The addition of a peripheral compartment resulted in a better model fit in comparison to only a central compartment (ΔOFV = − 50.4), with no additional benefit of two peripheral compartments.

### Stochastic model development

BSV estimates were identifiable for *V*_*1*_, *CL*_*B*_, *CL*_*F*_, *V*_*3*_, and *CL*_*3*_ with *η*-shrinkage below 10% (Table 2). Furthermore, BSV of *V*_*1*_ and *CL*_*B*_ and between *V*_*3*_ and *CL*_*3*_ were highly positively correlated. The proportional plus additive residual error model for both conjugated and free CPT best accounted for unexplained variability of the observed concentrations, with reported *ε*-shrinkage values of ≤ 10%.

**Table 2:** Final population pharmacokinetic parameter estimates

Conjugated CPT				Free CPT			
	Estimate (95% CI)	%RSE	Shr.		Estimate (95% CI)	%RSE	Shr.
Structural model							
${V}_{1} = {V}_{\mathrm{1,70}kg} \times {\left(\frac{\mathrm{BW}}{70\mathrm{kg}}\right)}^{1}$				${V}_{3} = {V}_{\mathrm{3,70kg}} \times {\left(\frac{\mathrm{BW}}{70\mathrm{kg}}\right)}^{1}$			
${V}_{\mathrm{1,70kg}} (L)$	3.16 (2.91–3.40)	4%		${V}_{\mathrm{3,70}kg} (L)$	21.1 (12.9–30.5)	22%	
${V}_{2} = {V}_{\mathrm{2,70kg}} \times {\left(\frac{\mathrm{BW}}{70\mathrm{kg}}\right)}^{1}$				${V}_{4} = {V}_{\mathrm{4,70kg}} \times {\left(\frac{\mathrm{BW}}{70\mathrm{kg}}\right)}^{1}$			
${V}_{\mathrm{2,70}kg} (L)$	2.09 (1.89–2.32)	5%		${V}_{\mathrm{4,70kg}} (L)$	19.4 (15.0-24.3)	13%	
${Q}_{1} = {Q}_{\mathrm{1,70kg}} \times {\left(\frac{\mathrm{BW}}{70\mathrm{kg}}\right)}^{0.75}$				${Q}_{3} = {Q}_{\mathrm{3,70kg}} \times {\left(\frac{\mathrm{BW}}{70\mathrm{kg}}\right)}^{0.75}$			
${Q}_{\mathrm{1,70kg}} (L/h)$	0.0482 (0.0381–0.0617)	12%		${Q}_{\mathrm{3,70kg}} (\mathrm{L}/\mathrm{h})$	25.6 (13.6–46.0)	33%	
${\mathrm{CL}}_{B} = {\mathrm{CL}}_{B,70\mathrm{kg}} \times {\left(\frac{\mathrm{BW}}{70\mathrm{kg}}\right)}^{0.75}$				${\mathrm{CL}}_{3} = {\mathrm{CL}}_{\mathrm{3,70kg}} \times {\left(\frac{\mathrm{BW}}{70\mathrm{kg}}\right)}^{0.75}$			
${\mathrm{CL}}_{B,70\mathrm{kg}} \, (L/h)$	0.0988 (0.0870–0.1129)	7%		${\mathrm{CL}}_{\mathrm{3,70kg}} (\mathrm{L}/\mathrm{h})$	0.874 (0.738–1.044)	9%	
${\mathrm{CL}}_{F} (\mathrm{L}/\mathrm{h})$	5.71 (3.89–7.85)	18%					
${t}_{1/2} (\mathrm{h})$	0.307 (0.265–0.356)	8%					
Random effects							
${\mathrm{BSV}}_{{V}_{1}}(\mathrm{CV}\%)$	18.1 (12.3–24.7)	36%	6%	${\mathrm{BSV}}_{{V}_{3}} (\mathrm{CV}\%)$	79.8 (56.8–117)	42%	5%
${\mathrm{BSV}}_{{V}_{1},{\mathrm{CL}}_{B}}(\mathrm{corr}. )$	0.918^#^			${\mathrm{BSV}}_{{V}_{3},{CL}_{3}} (\mathrm{corr}.)$	0.884^#^		
${\mathrm{BSV}}_{{\mathrm{CL}}_{B}}(\mathrm{CV}\%)$	33.5 (25.3–43.7)	29%	2%	${\mathrm{BSV}}_{{CL}_{3}} (\mathrm{CV}\%)$	42.2 (31.8–57.4)	33%	5%
${\mathrm{BSV}}_{{\mathrm{CL}}_{F}} (\mathrm{CV}\%)$	62.6 (44.2–86.4)	35%	5%				
Residual error							
${\mathrm{Bound} \,\mathrm{RE}}_{\mathrm{proportional}} (\%)$	12.3 (11.2–13.6)	10%	9%	${\mathrm{Free} \,\mathrm{RE}}_{\mathrm{proportional}} \,(\%)$	24.8 (22.3–28.1)	12%	10%
${\mathrm{Bound} \,\mathrm{RE}}_{\mathrm{additive}} (\mathrm{ng}/\mathrm{mL})$	5.07 (1.27–13.86)	64%	9%	${\mathrm{Free} \,\mathrm{RE}}_{\mathrm{additive}} (\mathrm{ng}/\mathrm{mL})$	0.396 (0.061–0.852)	53%	10%

95% CIs for parameter estimates were obtained via the 2.5 and 97.5% quantile estimates calculated during the SIR analysis

Conjugated CPT parameters: *V*_*1*_ central volume of distribution, *V*_*2*_ peripheral volume of distribution, *Q*_*1*_ inter–compartmental clearance (*V*_*1*_* − V*_*2*_), *CL*_B_ base conversion rate of conjugated CPT to free CPT (*V*_*1*_* – V*_*3*_), *CL*_*F*_ time-dependent rate conversion of conjugated CPT to free CPT (*V*_*1*_* – V*_*3*_), *t*_*1/2*_ half-life of first-order decay scalar term governing *CL*_*F*_-mediated CPT conversion

Free CPT parameters: *V*_*3*_ central volume of distribution, *V*_*4*_ peripheral volume of distribution, *Q*_*3*_ inter-compartmental clearance (*V*_*3*_* − V*_*4*_), *CL*_*3*_ clearance of free CPT from *V*_*3*_

Population estimates *V*_1,70 kg_, *V*_2,70 kg_, *Q*_1,70 kg_, CL_B,70 kg_, *V*_3,70 kg_, *V*_4,70 kg_, *Q*_3,70 kg_, and CL_3,70 kg_ correspond to a 70 kg subject and are adjusted to individual values using the corresponding parameter formulas

*CI* confidence interval, *%RSE* percent relative standard error, *Shr* shrinkage, *BW* body weight, *BSV* between-subject variability, *RE* residual error, *corr* correlation coefficient

^#^Correlation coefficient for specified covariance term is reported without 95% CI or %RSE

### Covariate model

Incorporation and refinement of clinically relevant covariates into the model were evaluated following the finalization of the stochastic model, starting with BW. CL_F_ and *t*_*1/2*_ were omitted from BW allometric scaling, as removal of the terms did not impact OFV and biologic plausibility was minimal; CL_F_ and its associated *t*_*1/2*_ are parameters influenced more so via physicochemical properties of the nanoparticle formulation in a pH neutral environment, and thus, neither were supposed to be influenced by BW. Free CPT allometric scaling was assumed to follow standard fixed allometric scaling terms, with exponents fixed to 1 and 0.75 for volume and clearance terms, respectively [23]. Alteration to allometric scaling of the conjugated CPT parameters was hypothesized based on proposed differences in nanoparticle size in comparison to small molecules. The exponents of the BW effect on *V*_*1*_, *V*_*2*_, *Q*_*1*_, and *CL*_*B*_ were estimated, yielding near equivalent values of 1.00 for each term but not providing a more optimal fit, thus justifying reversion to standard fixed exponents (i.e., 1.00 for volume of distribution terms, 0.75 for clearance terms), as described in Table 2. Sex and age did not significantly influence parameter estimates. Race and tumor type, though interesting parameters to consider, were not feasible to assess due to the small-sample size and the limited dataset. Additional physiologically based covariates that may influence free CPT PK, including serum albumin level and eGFR [24], were not reasonable to assess due to dataset limitations.

### Model evaluation and interpretation

**Fig. 2:**
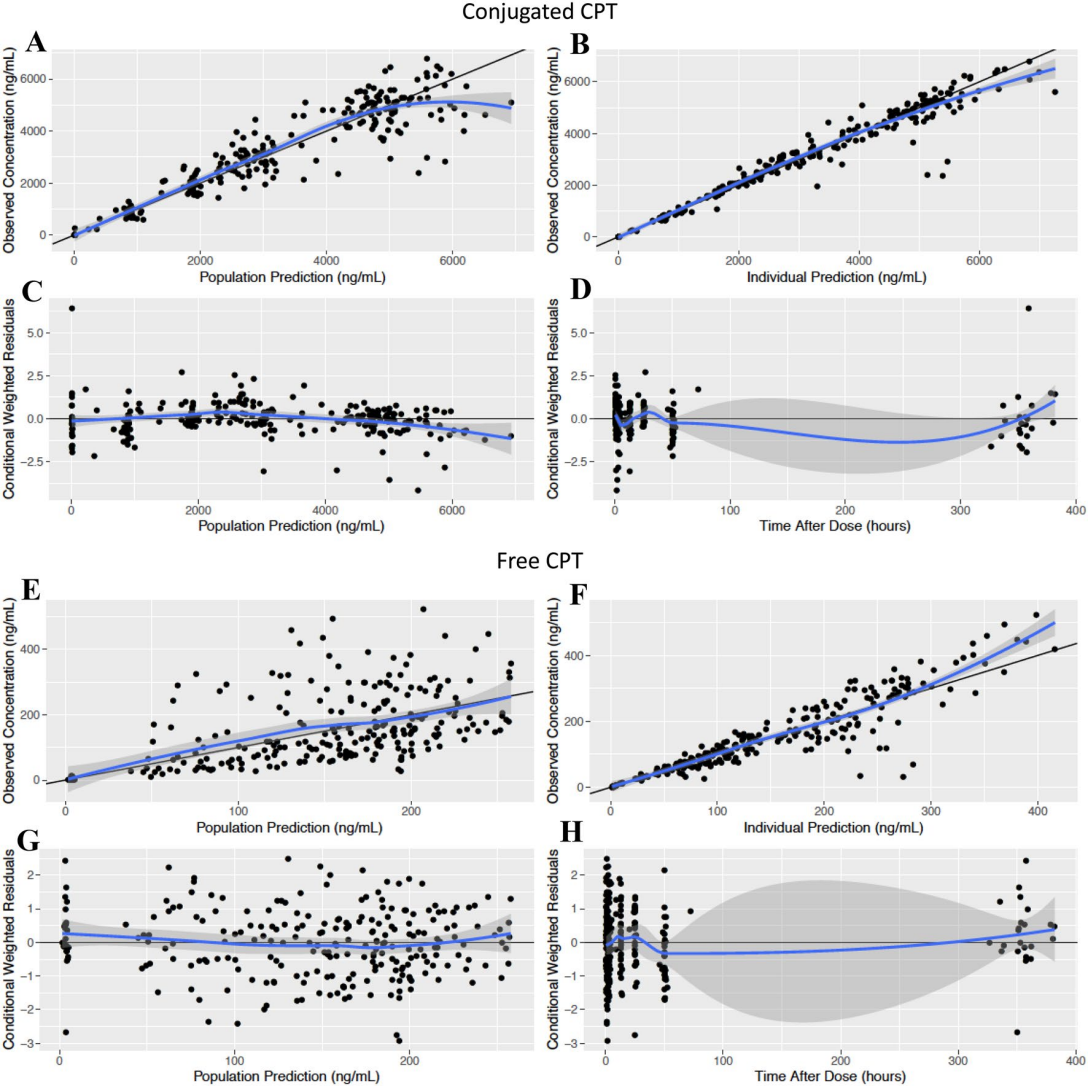
Goodness-of-fit plots for the pharmacokinetic model. Separate plots were generated for the evaluation of conjugated CPT (**a–d**) and free CPT (**e–h**). Plots include population prediction against observed data (**a, e**), individual prediction against observed data (**b, f**), conditional weighted residuals against population prediction (**c, g**), and conditional weighted residuals against time after dose (**d, h**). The solid black lines represent either the line of unity (**a, b, e, f**) or the zero line (**c, d, g, h**). Solid blue lines represent the local regression fit of the values

**Fig. 3:**
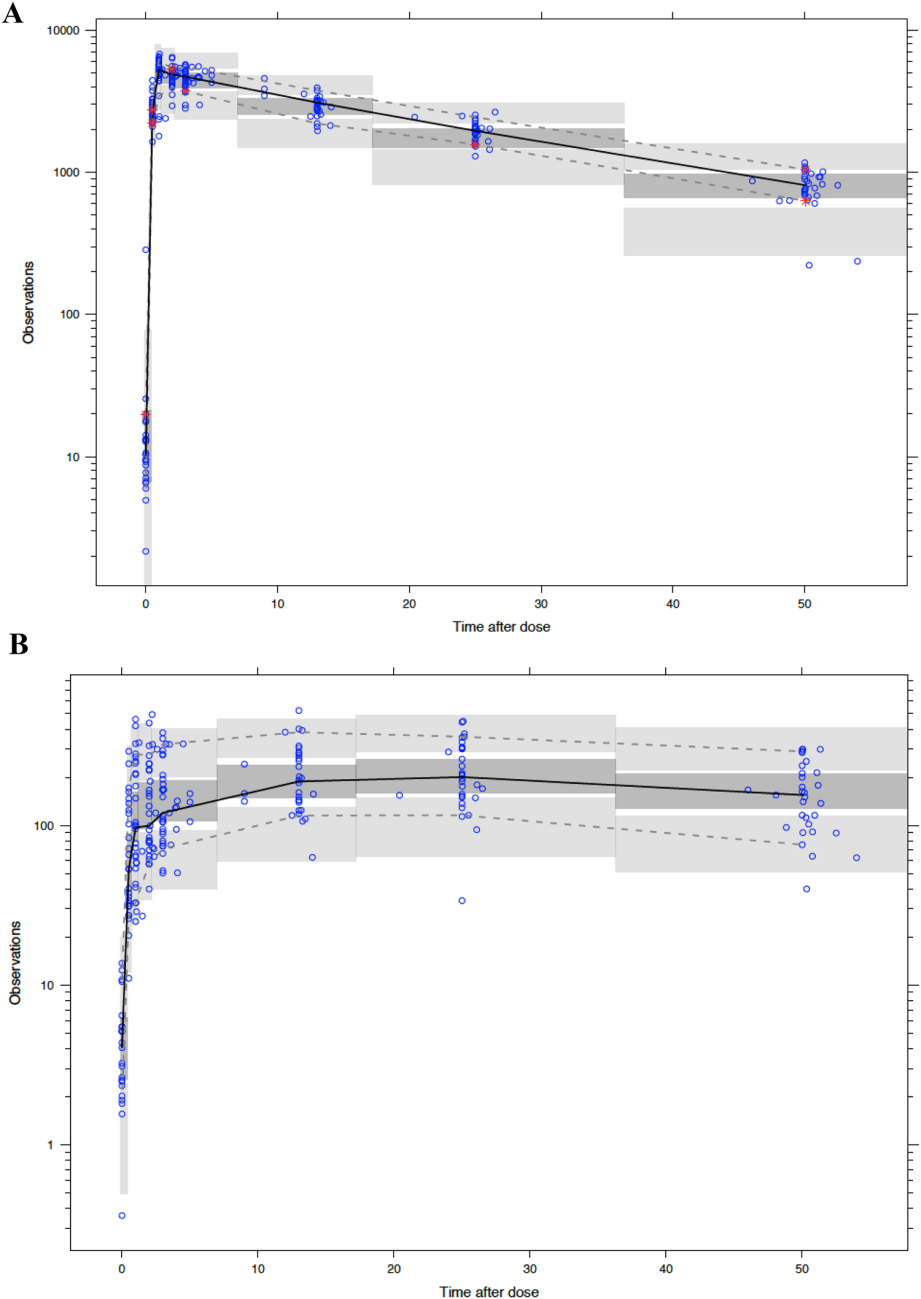
Visual predictive checks (VPCs) of conjugated (**a**) and free CPT (**b**) for timepoints up to ~ 50 h post-dose of NLG207. “Observations” are reported in units of ng/mL using log scale axis, and “time after dose” is reported in hours. Solid black lines depict the observed median and dashed lines represent the 2.5% and 97.5% percentile concentrations. 95% prediction intervals of the simulated mean and the 2.5 and 97.5% percentiles are represented by dark- and light-gray areas, respectively. Round dots represent observations and asterisks highlight observed percentiles outside of the prediction area

Parameter estimates and respective 95% confidence intervals (CI) of the final model are summarized in Table 2. All parameters were estimated with good to reasonable precision and verified identifiability of all parameters included in the model. Population and individual predictions were consistent with the observed data for both conjugated and free CPT, as shown via the GOF plots (Fig. 2). Conditional weighted residuals were symmetrically distributed in plots stratified by model prediction and time, consistent with minimal bias based on these parameters. VPCs confirmed that the observed data were consistent with simulated observations generated from the model (Fig. 3, S1). Inspection of the data is consistent with the formulation retaining a significant portion of total CPT during the first 48 h post-dose, as shown via the significantly higher exposure of conjugated CPT in comparison to free CPT and the delayed maximum concentration (*C*_max_) of free CPT.

## Discussion

To our knowledge, this is the first popPK model describing NLG207 pharmacokinetics beyond non-compartmental analysis (NCA). A critical objective of this model was to combine both conjugated and free CPT pharmacokinetics into a single model to better characterize the NDC formulation mechanistically. The final model utilized a minimal patient population with diverse tumor types to describe conjugated and free CPT disposition, and, importantly, the release of CPT from NDCs.

A rapid spike in free CPT concentrations early post-administration of NLG207, an observation noted in the previously published NCA [14], provided rationale to pursue the population approach to better characterize CPT release. By implementing the composite clearance term to characterize CL_1_, we were able to estimate first-order release (CL_B_) for later timepoints while estimating faster release (CL_F_) that dissipates via a first-order decay with estimated half-life, *t*_*1/2*_. Though the *t*_*1/2*_ estimate would suggest minimal influence of CL_F_ on CPT release by 1.8 h (or ~ 6 half-lives), the relatively large estimate of CL_F_ results in near equal contribution of CL_B_ and CL_F_ at this timepoint; only by 4 h, or ~ 13 half-lives, does the influence of CL_F_ on CPT release become negligible. The calculated half-life associated with CPT release (i.e., CL_1_) thus ranges from 0.38 h immediately at start of infusion to 22 h at 4 h post-start of infusion through the remainder of the dosing cycle. Our hypothesis states the outer surfaces of nanoparticles are responsible for the initial rapid CPT release; self-folding properties of the co-polymer formulation leave a small fraction of CPT molecules exposed, enabling hydrolysis from NDCs in the presence of physiological plasma pH with possible influence of esterase activity. Direct in vitro incubation of the co-polymer lyophilized powder in human plasma resulted in a similarly rapid release half-life of 1.7 h [20]. Previous data have also suggested that influence of esterase activity provides a minor contribution to overall CPT release, as indicated by a release half-life of 26 h in pre-frozen serum [24], a value close to our CL_B_ estimate. It is reasonable to suggest both pH-dependent and esterase-mediated hydrolysis contribute to early release rates, before stabilization of NDCs in plasma enables a first-order process to fully dictate release of CPT.

The linear two-compartment model of conjugated CPT demonstrated significant retention of CPT in the NDC formulation. Volume of distribution estimates in central and peripheral compartments (*V*_*1*_ and *V*_*2*_, respectively) were relatively low, consistent with high concentrations in both defined compartments and the physical chemical properties of the drug similar to macromolecules (e.g., monoclonal antibodies) [25]. Similarly, the prior NCA reported a mean steady-state volume of distribution of 4.63 ± 1.07 L (*n* = 6) and 2.42 ± 0.7 (*n* = 36) for 12 and 15 mg/m^2^ doses, respectively [14]. The estimate of inter-compartmental clearance, *Q*_*1*_, suggests slow equilibration of the conjugated CPT compartments, further supporting long retention in the systemic circulation.

The peripheral compartment of conjugated CPT, though ideally to represent specifically uptake into tumor, is a composite compartment representative of all tissue uptake, including significant uptake in the kidney, liver, and bladder, as noted in preclinical models [26]. Generally, models of carrier-mediated agents incorporate uptake into these tissues while also including interactions with the mononuclear phagocyte system (e.g., uptake into peripheral blood mononuclear cells [PBMCs] and the spleen) [27]; recent evidence has suggested a limited role of phagocyte-mediated uptake with NLG207 [28]. Furthermore, prior studies have confirmed the limited uptake into adjacent healthy tissues, typically absent of fenestrations large enough for nanoparticle uptake [8]. A limitation to note is the inadequate ability to address CPT release in the peripheral compartments, as though tumor tissue is generally more acidic [29], highly perfused tissues (i.e., liver and kidneys) have near equivalent pH to plasma [30, 31]. NDCs not only stably retain the majority of CPT, but also confine CPT in the plasma circulation and highly perfused tissues, permitting uptake only into tissues with appropriate size exclusion properties.

In comparison to conjugated CPT, the two-compartment free CPT model generated higher volume of distribution (*V*_*3*_ and *V*_*4*_) and inter-compartmental clearance (*Q*_*3*_) estimates, as expected for a small molecule. Mean central and peripheral compartment volumes of distribution for the free CPT model were 6.7- and 9.3-fold higher, respectively, in comparison to the corresponding estimates for conjugated CPT. Pharmacokinetic parameters were not routinely obtained via NCA during the time of initial CPT clinical trials; however, reported plasma concentration–time curves from four patients receiving large doses of free CPT suggests an approximate volume of distribution of 5–6 L [32]. Estimates of free CPT inter-compartmental clearance describe fast equilibration (half-life of ~ 16 min) of the central and peripheral compartments. Clearance of free CPT from the central compartment was slow by comparison, with a terminal half-life of 32.4 h. The aforementioned early CPT trial suggested a similar relationship between clearance terms, as equilibration and terminal half-lives of four patients ranged from 18 to 70 min and 10.8 to 19.6 h, respectively [32]. A two-compartment model describing topotecan popPK estimated a similar central volume of distribution to free CPT, but higher peripheral volume of distribution and faster clearances [33].

With respect to free CPT input, we also considered the possibility that a fraction of CPT would be released in the IV bag prior to administration, however, such models were not identifiable. Nonetheless, assuming that 100% of CPT was retained within the nanoparticle formulation prior to infusion was justified based on the reconstitution procedure for NLG207; reconstituted of NLG207 in sterile water for injection (SWFI) is added to dextrose 5% in water (D5W), which, per manufacturer label [34], is noted to have a pH of 4.3 and thus negligible release of CPT prior to infusion [20].

Allometrically scaling all parameters (excluding CL_F_ and *t*_*1/2*_) using BW adequately accounted for differences in body size, as the effect of sex did not appreciably contribute to a better model fit. The use of BW, though presenting with a similar fit to the BSA-adjusted model, was preferred given the evidence supporting allometric theory [35]. Incorporation of BSV revealed significant differences in parameter estimations of CL_B_, CL_F_, CL_3_, and, most notably, *V*_*3*._ Incorporation of BW only lead to an appreciable decrease in the BSV of *V*_*1*_, resulting in ~ 10% of the variability being explained by BW. No other significant covariates were identified in the present study.

Limitations of the present model are attributable to the patient population, sample collection, and parameter identifiability. Additional covariates worthy of exploration, including tumor type and compromised renal function, were not feasible given the small patient sample size (*n* = 27). The mechanistic effect of plasma albumin and LDL on free CPT and nanoparticle unfolding, respectively, also represent potential avenues for further model evaluation [24, 36]. Most notably, the model was unable to incorporate an estimate specifically characterizing renally cleared conjugated CPT during the first 24–48 h post-infusion, accounting for approximately 16% of the total dose in prior study [14, 24]. Separation of the typical 4–5 strands comprising a single NLG207 nanoparticle reduces particle size [8], thereby increasing the likelihood of glomerular filtration and likely contributing to observed conjugated CPT in urine. Accurate characterization of conjugated CPT renal clearance while accounting for nanoparticle unfolding would require incorporation of serial urine sample collection into the model.

The resulting model reinforced critical aspects of the NDC formulation, while providing an enhanced understanding of CPT release. Conjugated CPT PK had a small volume of distribution, low distribution clearance, and low final central clearance, characteristics consistent with the nanoparticle behavior of FDA-approved liposomal formulations (e.g., irinotecan, doxorubicin, and daunorubicin) [37–39]. A similar model characterizing liposomal irinotecan popPK utilized a clearance term consistent with saturable elimination (as implemented via the Michaelis-Menten equation) to describe the release of irinotecan from liposomes in plasma. In comparison to NLG207 PK, high initial release of irinotecan from liposomes is not present and free irinotecan exposure is considerably less, as indicated via the free drug central volume of distribution estimate of 401 L [37]. Properties unique to NLG207, including co-polymer self-assembly and pH-dependent release, delineate differences in free drug exposure from that of liposomal formulations; the composite clearance term for CPT release further emphasizes these differences, while also aiding mechanistic interpretation.

## Conclusion

NLG207 pharmacokinetics was best described using a harmonized model, combining separate two-compartment models of nanoparticle-bound and free CPT. Transfer between these model components incorporated two clearance terms to characterize rapid CPT release early post-dose and gradual conversion to a stable first-order rate of CPT release from the nanoparticle formulation. Allometric scaling based on body weight also accounted for between-subject variability. The current analysis of this small patient population provides a strong foundation for future study as a potential predictive tool with the availability of data from a larger sample size.

## Electronic supplementary material

Below is the link to the electronic supplementary material.

Supplementary file1 (DOCX 174 kb)
