# Supplementary material for: Population pharmacokinetic analysis of nanoparticle-bound and free camptothecin after administration of NLG207 in adults with advanced solid tumors
Source: Cancer Chemother Pharmacol. 2020 Sep 8;86(4):475–86. doi: 10.1007/s00280-020-04134-9 (PMC7515962; doi:10.1007/s00280-020-04134-9)
Supplement: Supplementary file 1 — Supplementary file1 (DOCX 174 kb) [file 280_2020_4134_MOESM1_ESM.docx]

**Supplemental Figure 1**

Visual Predictive Checks (VPCs) of Conjugated (**A)** and Free CPT (**B**) for timepoints up to ~360 hours post dose of NLG207. “Observations” are reported in units of ng/mL using log scale axis, and “time after dose” is reported in hours. Solid black lines depict the observed median and dashed lines represent the 2.5% and 97.5% percentile concentrations. 95% prediction intervals of the simulated mean and the 2.5 and 97.5% percentiles are represented by dark- and light-grey areas, respectively. Round dots represent observations and asterisks highlight observed percentiles outside of the prediction area.
